# Randomised controlled trial of the clinical and cost effectiveness of a specialist team for managing refractory unipolar depressive disorder

**DOI:** 10.1186/1471-244X-10-100

**Published:** 2010-11-29

**Authors:** Richard Morriss, Sarah Marttunnen, Anne Garland, Neil Nixon, Ruth McDonald, Tim Sweeney, Heather Flambert, Richard Fox, Catherine Kaylor-Hughes, Marilyn James, Min Yang

**Affiliations:** 1 School of Community Health Sciences, Division of Psychiatry and Institute of Mental Health, University of Nottingham, B Floor, Sir Colin Campbell Building, Triumph Road, Nottingham, NG7 2TU, UK; 2 Institute of Mental Health, University of Nottingham, Nottingham, UK; 3 Institute of Mental Health, Nottinghamshire Healthcare Trust, Nottingham, UK; 4 Institute of Mental Health and Business School, University of Nottingham, Nottingham, UK; 5 Institute of Mental Health and School of Social Policy, Sociology and Law, University of Nottingham, Nottingham, UK; 6 Institute of Mental Health and School of Community Health Sciences, University of Nottingham, Nottingham, UK

## Abstract

**Background:**

Around 40 per cent of patients with unipolar depressive disorder who are treated in secondary care mental health services do not respond to first or second line treatments for depression. Such patients have 20 times the suicide rate of the general population and treatment response becomes harder to achieve and sustain the longer they remain depressed. Despite this there are no randomised controlled trials of community based service delivery interventions delivering both algorithm based pharmacotherapy and psychotherapy for patients with chronic depressive disorder in secondary care mental health services who remain moderately or severely depressed after six months treatment. Without such trials evidence based guidelines on services for such patients cannot be derived.

**Methods/design:**

Single blind individually randomised controlled trial of a specialist depression disorder team (psychiatrist and psychotherapist jointly assessing and providing algorithm based drug and psychological treatment) versus usual secondary care treatment. We will recruit 174 patients with unipolar depressive disorder in secondary mental health services with a Hamilton Depression Rating Scale (HDRS) score ≥ 16 and global assessment of function (GAF) ≤ 60 after ≥ 6 months treatment. The primary outcome measures will be the HDRS and GAF supplemented by economic analysis incuding the EQ5 D and analysis of barriers to care, implementation and the process of care. Audits to benchmark both treatment arms against national standards of care will aid the interpretation of the results of the study.

**Discussion:**

This trial will be the first to assess the effectiveness and implementation of a community based specialist depression disorder team. The study has been specially designed as part of the CLAHRC Nottinghamshire, Derbyshire and Lincolnshire joint collaboration between university, health and social care organisations to provide information of direct relevance to decisions on commissioning, service provision and implementation.

**Trial registration:**

Clinical trials.gov identifier NCT01047124

## Background

By 2020, unipolar depressive disorder is projected to be the second leading cause of disability adjusted life years in the world [1], and with anxiety accounts for one per cent of the whole gross national product of a wealthy country like United Kingdom [2]. Depressive disorder is associated with significant functional impairment that can be restored following effective treatment [3]. Depressive disorder is persistent [4], possibly due to the fact that people with depression often do not seek treatment following relapse; when they do, it is rarely effective [5]. A longitudinal pattern of frequent recurrences with increasing severity can occur which leads to social damage and possible neurobiological changes which may be difficult to reverse [4]. Moreover suicide after unipolar depression accounts for 0.7% deaths [6]. Patients with chronic unipolar mood disorder that has been diagnosed by health services have a standardised mortality ratio for suicide around 20 [7], constituting a high risk group for suicide already identified by mental health services.

While there is plenty of research showing the short-term effectiveness of antidepressant medication and psychological treatments such as cognitive behaviour therapy, there are few randomised controlled trials of service interventions for depressive disorders that do not respond to first-line or second-line interventions. As a result treatment guidelines refer to the need to consult a specialist in the assessment and treatment of mood disorders [8] but are not specific in their recommendation about the nature of such an intervention.

Previous research gives some indication of what might be achieved. The influential STAR*D project carried out at 41 service settings in the US involving 3,671 patients with non-psychotic unipolar major depressive disorder demonstrated that with 1 to 4 different acute treatments lasting at least 14 weeks, 67 percent of patients achieved remission over one year [9]. NICE Guidelines for depression advocate a combination of antidepressant medication and cognitive therapy for severe and chronic depression [8]. In a meta-analysis of patients with severe recurrent depressive disorders the overall response rate to cognitive behaviour therapy (CBT) or interpersonal psychotherapy combined with antidepressant management (ADM) was three times higher (63%) than to brief psychotherapy alone (20%) [10]. The combination of ADM and CT (45% remission) was also more effective than ADM (29% remission) in a RCT of 158 participants with residual treatment-refractory depressive symptoms at 18 months [11]. These differences in outcome persisted for up to 5 years [12]. Overall, the combination of individually tailored antidepressant treatment followed by augmentation strategies or combined with cognitive therapy that follow algorithms of evidence based research appear to be the gold standard of treatment for depression [8,13]. An active, coordinated and thoughtful approach is necessary when treating chronic and severe depression [4].

There is evidence that many patients with unipolar depression in secondary care mental health services may not receive such an approach [5]. In STAR*D where treatment was delivered under optimal conditions, 40 per cent of patients failed to respond to first or second line treatment s for depression [9]. When both antidepressant medication and cognitive therapy approaches are applied in the same patient, they may not be effective if the timing of the interventions is not complementary. For instance, a patient may be too sedated from drug treatment to attend properly to cognitive therapy or drug treatment is dismissed as ineffective before issues surrounding medication adherence are addressed through cognitive therapy or psychoeducation. Therefore, a co-ordinated approach involving joint assessment and management of patients who have not responded to first-line and second-line treatment approaches for depressive disorder in secondary care services is required. The addition of psychotherapy to treatment as usual in a mixed group of mental health patients who had not responded to first or second line treatment was seen to be cost effective [14]. However, this study did not explicitly examine patients with depressive disorder and the form of psychotherapy that was used is neither widely available nor tested specifically in patients with unipolar depression. A small RCT also demonstrated the effectiveness of in-patient interpersonal psychotherapy with pharmacotherapy over usual in-patient care for patients with chronic major depressive disorder [15]. There is a considerable amount of trial evidence for stepped care interventions for primary care depressive disorder where care is coordinated and the nature of the intervention is tailored to the individual [16], and also some evidence for out-patient algorithm based care mostly involving medication for major depression [9,17]. However, to our knowledge there is no previous randomised controlled trial examining the clinical and cost effectiveness of a community based specialist depression disorders team offering time-limited algorithm based pharmacotherapy and psychotherapy to patients with unipolar depressive disorder who remain moderately or severely depressed after six months treatment by secondary care mental health services.

## Methods/Design

### Objectives

1. To determine whether a community based specialist depression disorder team, offering time-limited algorithm based pharmacotherapy and psychotherapy, to patients with unipolar depressive disorder who remain moderately or severely depressed after six months treatment for depression is more clinically and cost effective than continuing treatment from their continuing care teams in the secondary care adult mental health service.

2. To identify barriers, drivers and important therapeutic constituents of clinical care that was effective in either the specialist depression disorder or continuing care teams.

### Design

**Figure 1:**
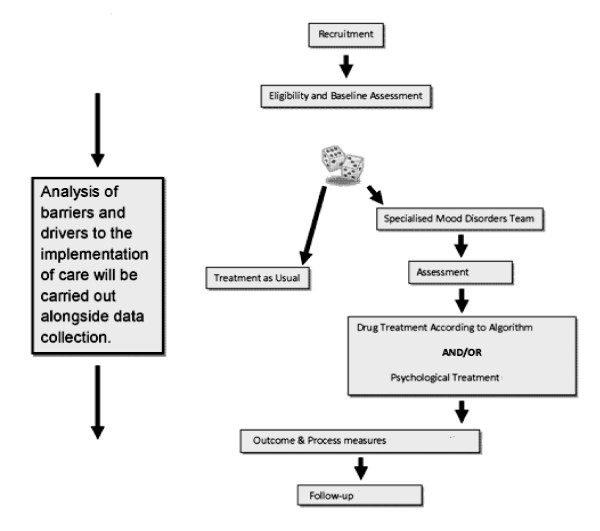
**Flow of patients in the randomised controlled trial of Specialist Mood Disorder Team versus usual care**.

A pragmatic single blind randomised controlled trial (RCT) of a specialist depression disorder intervention versus treatment as usual will be conducted. Participants will be individually randomised with stratification by mental health trust and allocated to each group on a one to one basis. Eligible participants will be followed for 24 months. The primary outcome will be observer rated depressive symptoms over the first 12 months but further analysis will explore if any differences in outcome are maintained at 24 months, 12 months after the patient has been discharged from the specialist depression disorder team. Figure 1 shows the overall design of the study.

The RCT forms part of the Nottinghamshire Derbyshire and Lincolnshire Collaboration for Leadership in Applied Health Research and Care (CLAHRC NDL), an applied health services research centre funded by the Department of Health, the University of Nottingham and nine health and social organisations in three English counties [18]. It focuses on service innovation and implementation of research as well as clinical and cost effectiveness of interventions that are perceived by the local health services to be a high priority.

Unlike traditional RCTs, which favour a small number of clinical outcomes, service delivery studies of complex interventions require multiple outcomes [19]. Service delivery studies are interested in a range of equally important outcomes including clinical outcome, cost effectiveness, access to services, burden on staff and risk of serious adverse events. Nevertheless, multiple outcome measures can lead to false positive conclusions about the effectiveness of a treatment so a primary outcome variable (change in depressive symptoms) is specified. In order to interpret the results of such a RCT, and identify important processes and context variables required for replication, a range of outcomes and process variables is necessary [19]. Therefore, alongside the RCT an implementation analysis of barriers and drivers to effective care using largely qualitative methods is performed. An audit will be performed of the standard of care delivered to the patient by secondary mental health care before and after entry to the study using NICE Guidelines for depression, the standard of care that is expected to be delivered in England and Wales [8,20]. The standard of care provided by the continuing treatment teams in the secondary care mental health services is important to measure in order to interpret the results of the RCT; if the standard of care is high in usual care, there may be ceiling effects operating even if the specialist depression disorder team is effective, but if usual care is poor then the specialist depression disorder team may be effective merely because it is providing an acceptable standard of care. In the latter case, the implication might be that better training and support for existing staff is required rather than the formation of a specialist depression disorder team.

A further audit will carried out to examine the representativeness of the sample who enter the RCT in terms of both demographic characteristics and the treatments they have received to examine the generalisability of the results. Finally, for the purposes of replication, both descriptive statistics and analytical methods using a range qualitative approaches and quantitative process measures will be employed. Unlike simple drug and psychological treatment interventions, services can vary at many levels (organisationally, clinician-patient, patient sample, nature of treatment) so it is important to specify the important constituents of an effective intervention and the processes that are necessary to achieve a successful outcome [19].

### Sample and inclusion/exclusion criteria

The intention to participants in secondary care mental health services who continue to suffer from moderate or severe depression despite continuous treatment for at least six months. Such patients may receive in addition to secondary care mental health services, interventions for depression provided by primary care, voluntary and private sectors. Eligible patients will be under the care of a secondary care community mental health team or out-patient services provided by three mental health trusts in England. The Structured Clinical Interview for DSM-IV Axis 1 Disorders [21] will be used to describe patients' symptom profile at baseline. The pragmatic nature of this study requires that inclusion/exclusion criteria must reflect everyday criteria that NHS clinicians use and would be used by a specialist depression disorders team [22]. Inclusion criteria are:

• The responsible medical officer or care coordinator leading the patient to be suffering from primary unipolar depression which is not a consequence of having another axis 1 or 2 psychiatric disorder;

• Age over 18 years;

• Able and willing to give oral and written informed consent to participate in the study;

• From the date of first assessment by a health professional working within the index mental health trust, primary care trust or third sector, they must have been offered or received direct and continuous care from one or more health professionals in the preceding 6 months. They must currently be under the care of a secondary care mental health team;

• Meets NICE criteria for moderate depression (five out of nine symptoms of depression [8]); has a Hamilton Depression Rating Scale of at least 16, indicating at least moderate severity depression [23]; and score 60 or less on the Global Assessment of Functioning Scale implying at least moderate impairment in social or occupational function and/or moderate symptoms of depression [24].

Exclusion criteria are:

• Is receiving emergency care for suicide risk, risk of severe neglect or homicide risk; however, patients will not be excluded because of such risk provided the risk is adequately contained within their current care setting and the primary medical responsibility for care remains with the referring team;

• Does not speak fluent English;

• Is pregnant

• Unipolar depression is secondary to a primary psychiatric or medical disorder.

However, patients with bipolar disorder which has not been diagnosed by the primary clinical team but detected at baseline in the course of the research will not be excluded because in NHS clinical practice they would be looked after by specialist depression disorders and usual care teams.

### Interventions

#### Specialist Depression Disorder Team (SDDT)

The SDDT will consist of a team of psychiatrists and cognitive behaviour therapists who will work together. All of them are experienced clinicians who treat depression as part of the secondary health care service. They will take a stepped care approach as outlined by the NICE guidelines. A key feature of the specialist mood disorder team is that a psychiatrist and a cognitive behaviour therapist will jointly assess the patient and agree upon a joint formulation of the patient's problems focusing on maintaining factors for the depression. They will then agree upon a joint management plan and review progress during treatment so that the two management approaches complement each other. The psychiatrist will follow a treatment algorithm derived from NICE guidelines for drug treatment of depression, British Association of Psychopharmacology [13] and findings from the STAR*D project [9] after an assessment and review of recent treatment (copy available from the authors). This treatment algorithm is a guide to be interpreted in the light of the assessment made by the psychiatrist and the patient's treatment history. All participants in the intervention group will receive at least a psychoeducation approach incorporating cognitive behaviour therapy (CBT) techniques. However, some patients will receive mindfulness based CBT [25], standard CBT [26] or compassionate mind based CBT [27] according to the therapist's assessment. The psychiatrist and cognitive behaviour therapist will also consider social approaches and when relevant consult professionals providing social care to complement pharmacological and psychological interventions. Physical treatments such as electroconvulsive therapy will not be employed by the SDDT.

Participants being treated by the SDDT will each receive a unique treatment plan tailored to their specific needs. They may receive up to three or four different drug treatment approaches and two different psychological treatment approaches over the 12 month intervention period. The nature, time taken, form and content of the assessments, supervision, decisions and discussions between different members of the specialist depression disorder team will be logged and recorded.

At the end of the study participants will be re-integrated into their usual care team. Any new medications started will be continued with usual care but any psychological treatment will be completed.

#### Treatment as usual

Treatment as usual will be provided by the clinical team that referred the patient to the study and will be unconstrained other than it will not be provided by the psychiatrists in the SDDT. Economic data collection and an audit of case notes will provide information on the interventions given during treatment as usual within the trial itself.

### Outcomes

**Primary outcome **measures are:

1. Longitudinal change in the 17-item observer rated Hamilton Depression Rating Scale (HDRS) [23,28] from baseline to 6 and 12 months as well as follow up assessments at 18 and 24 months. The primary analysis will be change over the baseline to 6 and 12 months. The purpose of the analysis at 18 and 24 months is to determine if any change is maintained once the patient has been discharged from the specialist depression disorder team.

2. Change in global assessment of function [24] from baseline to 6 and 12 months as well as follow up assessments at 18 and 24 months.

**Secondary outcome **MEASURES ARE:

1. Change in self-rated depression: a) self-rated Beck Depression Inventory version 1 [29], a measure of cognitive symptoms of depression from baseline to 3, 6, 9, 12, 18 and 24 months; b) Personal Health Questionnaire [30] from baseline to 3, 6, 9, 12, 18 and 24 months, rating of depression severity according to DSM-IV criteria; c) Quick Inventory for Depressive Symptomatology self rated version [31] from 3, 6, 9, 12, 18 and 24 months, a 16-item screening/diagnostic questionnaire rating depression severity according to DSM-IV criteria. The last scale has the best established psychometric data for remission compared to the Hamilton Depression Rating Scale. The other two scales are widely used in clinical practice by general practitioners and psychotherapy services in England and Wales.

2. Euroqol 5 D [32] as a measure of quality of life and costs from health and social care and society perspectives measured at 6, 12, 18 and 24 months. Use of the EQ5 D will enable utility score sto be gained from the patients that may then be used in a cost utility analysis

3. Change in social adjustment [33], an assessment of social and occupational functioning from baseline to 6, 12, 18 and 24 months.

4. Patient satisfaction and relationship with the clinician(s) on a four part 9-item questionnaire based on two other questionnaires used frequently in depression studies: the Patient Satisfaction Questionnaire [34] and the Patient Doctor Relationship Questionnaire [35]. These will be measured at 6, 12, 18 and 24 months.

**Process measures**, which will not be utilised to determine the effectiveness of the interventions, but will be used to understand the processes that are taking place:

1. At baseline care received will be audited against standards of care outlined in NICE Guidelines [8,20] according to a 4-point scale (1 = not followed NICE Guideline, 2 = followed NICE Guideline< 50% of the time, 3 = followed NICE Guideline > 50% of the time, 4 = fully followed NICE Guideline) by an independent clinical expert. This assessment will then be applied to each three month block of care during the 12 month follow-up.

2. The number of patients with unrecognised axis 1 and 2 psychopathology [24] and medical co-morbidity will be recorded by the research team and also audited against treatment notes to determine if the specialist team is more accurate in terms of diagnosis.

3. At baseline life events and difficulties in the preceding 6 months and at 6, 12, 18 and 24 months after baseline will be recorded using the Brugha 12-item life event checklist [36]. The recognition or not of these life events and difficulties according to case notes will be audited in the two treatment groups.

4. At baseline social support in the preceding 6 months and at 6, 12, 18 and 24 months after baseline will be recorded using the 3 item social support and social network measure [37], which examines such support networks. The recognition or not of this social support according to case notes will be audited in the two treatment groups.

5. Adherence to medication [38], assessed at 6 and 12 months.

Quality of relationship between the patient and any secondary care mental health professional they have seen on a planned ongoing basis to manage their depression on a 9 item patient rated scale [35] assessed at baseline, 6 and 12 months.

6. Brief self-rated measures demonstrating the suitability of the cognitive therapy offered to the patient's needs: a) 18-item Others as Shamer Scale [39]; b) 22-item Forms of Self-criticising/attacking and Self-reassuring Scale [40]; c) 26-item How I act towards myself in difficult times scale [41]; d) 16-item Social Comparison Scale [42]; e) 16-item Entrapment Scale [43]; f) 16-item Defeat Scale [43]; g) 39-item 5 Facet Mindfulness Questionnaire [44].

7. At baseline, 6, 12, 18 and 24 months, participants' overall ruminative processes will be captured using the Rumination Scale [45]. Symptoms of rumination can predict vulnerability to depression, particularly relapse. As psychological treatments in the specialist mood team will aim to modify the ruminative process, this questionnaire will inform us whether this aim is being achieved.

8. At baseline, 6, 12, 18 and 24 months, participants' overall tendencies to avoid thinking about painful emotional issues will be measured using The Acceptance and Action Questionnaire-1 [46]. Patients who score highly on this measure are likely to need more preparation before they can undertake psychological treatment.

All measures will be assessed at baseline, 6, 12, 18 and 24 months face-to-face by the research associate (unless otherwise stated). At 3 and 9 months the Beck Depression Inventory, the PHQ-9, the QIDS-SR and a questionnaire version of the health economics interview will be mailed to all participants.

### Sample size and justification

Sample size calculation was based on improvement in global assessment of severity in a study using a similar design, except that it employed a mixed diagnostic group rather than moderate to severe primary depressive disorder [14], 90% power, 2 tailed difference at 5% significance, 20% loss to follow up was 52 per treatment group (104 in total). However, this study did not employ an intention to treat analysis and there was a 30 percent loss to follow-up; therefore, the sample size has been inflated by a further 43 percent to 74 per group (148 in total). A further correction is to be made for the variability in the individual treatment from the SDDT and treatment as usual. A multiplicative correction factor to the sample size estimate of 1.18 calculated from [1 +rho*r/(1-rho)] [48] where rho is the intraclass correlation of 0.051[49] and r is the number of patients from each community mental health team (CMHT) per treatment arm (3-4). Therefore, the sample size is 87 per treatment group (174 in total). Sample size calculations were checked against a study of in-patient delivered combined psychotherapy and pharmacotherapy versus treatment as usual for patients with chronic depressive disorder [15]. The primary outcome variable was the 17-item Hamilton Depression Rating Scale (HDRS) and patients were followed up for 12 months. At baseline the combined treatment mean (sd) HDRS was 25.6 (4.4) and for clinical management it was 23.5 (4.8). At 12 months the HDRS score was 5.9 (5.1) in the combined treatment group and 11.3 (10.5) in the clinical management group (intention to treat analysis). Using a 2-tailed students t-test, 90% chance of detecting a difference at 0.05 level, and an effect size of 0.65, 51 patients per group are required (102 in total). If a 20 percent drop-out is assumed, then 122 patients (61 in each group) are required. Using the correction factor of 1.18 previously justified results in a sample size of 146 (73 in each group). In line with the more conservative estimate of the power of the study our aim is to recruit 87 patients per treatment group (174 in total).

### Randomisation

Once baseline assessments are completed by the research staff, patient details are sent to a Clinical Trials Unit (CTU) by the trial secretary. The treatment to which a patient is assigned is determined by a computer generated pseudo-random code using random permuted blocks of varying size, created by the CTU in accordance with their standard operating procedure and held on a secure server. Patients will be allocated with equal probability to each treatment arm with stratification by Trust. Allocation of the patients to a treatment arm is conveyed by the computer to the trial secretary who relays this information to a secretary supporting the SDDT and the referring clinician who will be expected to organise the patient's care if allocated to treatment as usual. Only the trial co-ordinator and trial secretary have password access to the randomisation data and research associates performing outcome assessments will not have access to the patient's health service records.

### Blinding

The research associate responsible for performing the baseline and outcome assessments will remain blind to randomisation until data collection has been completed. Any cases of unblinding are recorded. Researchers performing follow-up interviews will guess which group the participant has been randomised to at the end of 12 months treatment. At the end of the study, these guesses will be compared against chance.

### Statistical analysis

Statistical analysis for quantitative measures will be on the 'intention-to-treat' basis and carried out by the research team in two stages. At the first stage, analysis will be focused on process measures. Differences at each time point and in changing patterns over time between the two treatment groups and amongst clinical sites will be examined. Results from such analysis will help us to identify possible covariates or confounders at the individual level or clinical level that may need to be adjusted for when comparing the primary of secondary measures between treatment groups. Mechanism of missing data on major outcomes will be examined by sensitivity analysis, to inform adequate imputation procedures. At the second stage, the HDRS of the primary measure as well as multiple secondary measures will be analysed separately and jointly. As all measures are taken longitudinally, multilevel models for repeated measures will be used [49]. Data of patients who dropped out or not completed follow-up measures will be analysed in the manor of last-observation-carried-forward in multilevel modelling. The core model for each measure will be two-level with individuals as level 2 units and time occasions as level 1 units. If random effects or large variation among clinical sites are detected in the first stage analysis, the core model will be extended to three-level with clinical sites at the top of the hierarchy as level 3 units to account for random effects among clinics. For analysing multiple measures (or multiple end points) simultaneously to investigate global change or multi-dimensional change of the intervention effects, the core model will also be extended to a three-level structure with measures at the bottom of the data hierarchy. All models will estimate the mean changes of measures from the baseline to the later time points for each treatment group, and differences in such changes between groups by interaction terms between time and treatment group in models, adjusting for possible confounders or covariates. For continuous measures with reasonably symmetric distribution, ordinary multilevel models will be used in the analysis. Otherwise, data transformation before model fitting will be considered. For count data with a long tail in the distribution, multilevel Poisson models will be considered [50]. For ordinal measures, multilevel multinomial models will be considered [51]. Descriptive and simple statistical analysis will be performed in SPSS and MLwiN [52] will be used for multilevel models analysis.

### Health economics

We will ascertain health, social and personal costs and examine cost utility and cost effectiveness from health and social care, and societal perspectives. The aim is estimation in relation to NICE thresholds for cost effectiveness rather than significance testing [8]. At baseline, 6 and 12 months the research associate will interview patients using a modified version of the Client Service Receipt Inventory [53]. At 3 and 9 months a modified self-report version of the Client Service Receipt Inventory will be mailed out to patients. At baseline and 12 months the research associate will interview patients using a modified version of the Client Service Receipt Inventory [53]. This inventory records inputs given by family members as a result of the patient's depression, as well as the amount of time off work by the patient and any carer due to depression including the costs of such. Data on social security payments will also be collected by qualitative interview. Nationally applicable unit costs [54] will then be combined with the service user data to generate service costs. Medication costs will be obtained from the British National Formulary and hospital based costs will be obtained from NHS reference costs. The cost of the specialist intervention can be calculated with the help of diaries showing time spent by the different members of the team in delivering the work of the specialist mood disorders team together with these unit health costs. Interviews with treatment as usual teams will generate estimates of all hidden costs for team discussion and supervision that the patient will not be aware of. Costs of time off work will be calculated from the patient's own account of their salary and normal expectations of overtime and informal care to the depressed patient will be calculated at the commercial rate that a carer would have to be paid through an agency. Personal information that may identify the participant will not be collected during these questionnaires and qualitative interviews; therefore, confidentiality will be maintained.

### Implementation analysis

The implementation analysis will provide important information on the barriers and drivers to the delivery and implementation of care in both treatment arms. The implementation analysis will involve four interrelated approaches to data collection to map the implementation of the SDDT and compare this with usual care. In addition, data collection will also involve other stakeholders who have responsibility for delivering, managing or commissioning services as part of the process of usual care. These stakeholders will include individuals across the mental health care and primary care. The four interrelated approaches include documentary analysis [55,56], interviews [57], social network analysis [58] and observation [59].

#### Documentary analysis

Key documents relating to the implementation, delivery and ongoing commissioning of the project will be analysed to provide evidence of the challenges and facilitators faced in the implementation of the project. These may include pre-existing national treatment guidelines [8,10]; trust-level guidance on implementing treatment guidelines; documents produced by commissioners on service-level agreements with providers around service design; any available minutes from primary care and secondary care providers; and any guidelines produced for practitioners by professional associations such as the Royal College of Psychiatrists.

#### Interviews

Interviews with key stakeholders involved with the commissioning, management and delivery of care will address issues relating to the uptake of existing NICE Guidelines for depression and in the delivery of interventions by the SDDT. Stakeholders will be approached by diffusion fellows (clinical staff employed on the project for one day per week) and interviews will be qualitative in nature. We will interview individuals involved in the control and intervention arms of the trial including patients, psychiatrists, psychologists, psychotherapists, pharmacists, members of community mental health teams, general practitioners, and service commissioners. There is no specific inclusion/exclusion criteria for these individuals save that they are involved in the care of patients. The sample size for this analysis cannot be pre-determined as it will depend on the size of the network treating these individuals and the amount of variance in that treatment from site to site. Subject to their agreement, some of these individuals may be re-interviewed towards the end of the research, to discuss issues raised in their initial interviews and to reflect on changes in the field that have subsequently taken place. Information sheets outlining the study will be given to those who are interested in taking part in the implementation analysis and informed consent will be obtained prior to any interviews taking place.

#### Social Network Analysis

Each staff interviewee will be asked to complete an SNA pro forma giving details of the individuals with whom they interact on a regular basis to do with service care. SNA will enable the researchers to understand the network of individuals involved in the commissioning, management and delivery of care; and, to identify areas where relationships appear strong or form weaker interactions.

#### Observation

Researchers will attend meetings across the time scale of the trial in order to contextualise the understanding developed through interviews and SNA. These meetings will allow researchers to apprehend the challenges and facilitators to the implementation of the project. In addition to these four approaches carried out by the research team, stakeholders will be invited to keep reflective diaries on the issues they face when putting guidance into practice and to gather their ideas about the kinds of changes that might mitigate the difficulties to implementation.

### Results

The study is funded, has received ethics and research governance approval and is now recruiting participants.

## Discussion

The funding provided through CLAHRC NDL has provided a unique opportunity to carry out a RCT of a specialist depression disorder team compared to usual care across three health care organisations. There are no previous randomised controlled trials, partly because it is rarely possible to persuade a number of healthcare organisations to reorganise their services and provide the resources required to undertake such a trial. Without such RCTs it is not possible to develop evidence based guidelines on the organisation of services for patients with depression who remain moderately or severely depressed even after first and second line treatment from secondary mental health care services. The CLAHRC is able to do this because it provides the senior managerial, political, commissioning, clinical, academic and financial support to carry out such service redesign trial, including the input of multiple academic disciplines from psychiatry, nursing, psychology, medical statistics, health economics, sociologists and business management. It has benefited also from the advice and active involvement in recruitment of service users with chronic depression who are essential for a full understanding of the optimal delivery of services.

### Strengths

The main strength of the study is that it is a pragmatic randomised controlled trial designed to test two interventions as they would be delivered in routine clinical practice in England. Therefore these interventions are delivered by health service clinicians who already provide psychiatric and psychological treatments in the health service rather than specialist experts especially drafted into the study. The study uses inclusion/exclusion criteria that reflect the patients that a SDDT would treat if such a service it existed. Thus it would take only patients with depressive disorder who had failed to improve after a period of time in generic mental health services and it would not take patients who had persistently failed to attend generic mental health service treatment that had the capacity to treat the patient in their own home if necessary. The patients would also have to remain symptomatically and functionally moderately to severely impaired.

Unlike most traditional research the project was developed with the full involvement of higher management, commissioners, mental health service staff and service users which should help the study to be completed and the results to be properly considered for implementation. To achieve this, a broader range of outcomes than clinical effectiveness need to be measured. Therefore economic outcomes and implementation issues are being fully explored so that decisions can be made about the cost effectiveness of the SDDT and how it would work optimally in clinical practice. The latter requires qualitative approaches to identify barriers that are not immediately obvious such as attitudes and organisational issues, and also quantitative measures to track the process of care to ensure that the interventions are producing the clinical changes that would be anticipated. For instance if mindfulness CBT is employed then there should be predicted improvements in mindfulness [46] and rumination [47] in these patients compared to both their baseline and overall treatment as usual scores. Furthermore the representativeness of the patients in the RCT both in terms of sociodemographic characteristics and treatment received will be examined. The use of audit of treatment received versus NICE Guidelines before entry into the study and in the two year follow up period will aid the interpretation of the study by benchmarking care received against national guidelines [8,20].

### Weaknesses

A weakness of the current study design is that there is no previous pilot data which could be used to derive an effect size of treatment by a SDDT, to determine recruitment rates and throughput through the SDDT. Furthermore as the patients will be drawn from multiple clinical sources, variance in outcomes may be larger than we have estimated. As a result the study may be underpowered and unable to provide a definitive answer to whether a SDDT is more effective than treatment as usual although it will be able to provide estimates of effect size for future RCTs of this type of intervention in out-patient or community settings. Another weakness is that there is the potential for contamination between the treatment groups; as the SDDT treats more patients, then it may influence the treatment provided by other health professionals delivering treatment as usual. Therefore over the years of recruitment, treatment as usual may change as a direct influence of the trial and become closer to national guideline treatment so a true difference between the two groups will be difficult to show. The design does permit an assessment of whether treatment as usual has evolved over time through the benchmarking process against NICE Guidelines for Depression [8]. The trial will not permit a direct evaluation of the interventions themselves, only the sum of their effect when delivered as a service. Finally, the results may not be generalisable to other populations with different sociodemographic characteristics or where the standard of care might be better or worse than provided in mental health services in this study.

The proposed randomised controlled trial will be a pragmatic trial run under conditions that are as close as possible to clinical practice in the NHS within the confines of running a trial. The trial itself has therefore been designed deliberately in terms of inclusion/exclusion criteria, personnel delivering treatment and additional methodology such as economics, implementation analysis and audits to provide all the information required for service providers, service commissioners and researchers to make decisions on implementation of a SDDT or the organisation of care for people who remain moderately or severely depressed after six months or more secondary care treatment.

## Abbreviations

ADM: antidepressant medication; CBT: cognitive behaviour therapy; CLAHRC: Collaboration for Leadership in Applied Health Research and Care; CLAHRC NDL: Nottinghamshire Derbyshire and Lincolnshire Collaboration for Leadership in Applied Health Research and Care; CMHT: community mental health team; CTU: Clinical Trials Unit; EQ5D: Euroqol 5 D measure; GAF: Global Assessment of Functioning; HDRS: Hamilton Depression Rating Scale; NICE: National Institute for Clinical Excellence; RCT: randomised controlled trial; sd, standard deviation; SDDT: Specialist Depression Disorder Team; SPSS, Statistical Package for the Social Sciences; STAR*D: Sequenced Treatment Alternatives to Relieve Depression study.

## Competing interests

The authors declare that they have no competing interests.

## Authors' contributions

All authors contributed the design of the study, the study protocol and the writing up of the paper. All authors read and approved the final manuscript. RM and AG wrote the project application for funding.

## Pre-publication history

The pre-publication history for this paper can be accessed here:

http://www.biomedcentral.com/1471-244X/10/100/prepub
